# Cultural legacies, fire ecology, and environmental change in the Stone Country of Arnhem Land and Kakadu National Park, Australia

**DOI:** 10.1002/ece3.460

**Published:** 2013-01-04

**Authors:** Clay Trauernicht, Brett P Murphy, Natalia Tangalin, David M J S Bowman

**Affiliations:** 1 School of Plant Science, University of TasmaniaPrivate Bag 55, Hobart, TAS, 7001, Australia; 2 Geographic Information Science Centre of Excellence, South Dakota State University1021 Medary Ave, Wecota Hall Box 506B, Brookings, SD, 57007, USA; 3 School of Botany, University of MelbourneMelbourne, VIC, 3010, Australia; 4 National Tropical Botanical Garden3530 Papalina Rd, Kalaheo, HI, 96741, USA; 5 Botany Department, University of HawaiiHonolulu, HI, 98622, USA

**Keywords:** Aboriginal landscape burning, Coupled human natural systems, fire ecology, fire management, habitat heterogeneity, landscape history, plant community diversity, tropical savanna

## Abstract

We use the fire ecology and biogeographical patterns of *Callitris intratropica*, a fire-sensitive conifer, and the Asian water buffalo (*Bubalus bubalis*), an introduced mega-herbivore, to examine the hypothesis that the continuation of Aboriginal burning and cultural integration of buffalo contribute to greater savanna heterogeneity and diversity in central Arnhem Land (CAL) than Kakadu National Park (KNP). The ‘Stone Country’ of the Arnhem Plateau, extending from KNP to CAL, is a globally renowned social–ecological system, managed for millennia by Bininj-Kunwok Aboriginal clans. Regional species declines have been attributed to the cessation of patchy burning by Aborigines. Whereas the KNP Stone Country is a modern wilderness, managed through prescribed burning and buffalo eradication, CAL remains a stronghold for Aboriginal management where buffalo have been culturally integrated. We surveyed the plant community and the presence of buffalo tracks among intact and fire-damaged *C. intratropica* groves and the savanna matrix in KNP and CAL. Aerial surveys of *C. intratropica* grove condition were used to examine the composition of savanna vegetation across the Stone Country. The plant community in intact *C. intratropica* groves had higher stem counts of shrubs and small trees and higher proportions of fire-sensitive plant species than degraded groves and the savanna matrix. A higher proportion of intact *C. intratropica* groves in CAL therefore indicated greater gamma diversity and habitat heterogeneity than the KNP Stone Country. Interactions among buffalo, fire, and *C. intratropica* suggested that buffalo also contributed to these patterns. Our results suggest linkages between ecological and cultural integrity at broad spatial scales across a complex landscape. Buffalo may provide a tool for mitigating destructive fires; however, their interactions require further study. Sustainability in the Stone Country depends upon adaptive management that rehabilitates the coupling of indigenous culture, disturbance, and natural resources.

## Introduction

Fire is an ancient and pervasive disturbance among terrestrial ecosystems yet poses formidable challenges for conservation and socio-economic development (Bowman et al. 2009). Although climate undoubtedly affects fire regime dynamics (Marlon et al. 2008; Mooney et al. 2011), the relative influence of human management versus biophysical constraints on fire activity is still debated. Fire has been a fundamental aspect of human culture throughout our history (Pyne 1997; Bowman et al. 2011), and there is growing evidence that anthropogenic burning, even among pre-industrial societies, has had ecosystem-scale effects worldwide (Nowacki and Abrams 2008; Fletcher and Thomas 2010; Archibald et al. 2012). Indeed, the current challenges facing many flammable landscapes (e.g., increases in fire intensity and extent, diversity declines) are associated with the breakdown, via colonization and dispossession, of culturally integrated, indigenous approaches to fire management (Russell-Smith et al. 1997; Laris 2002; Rodriguez 2007). Coupled social–ecological systems can provide critical insight into how the changing human dimension of fire affects the sustainability of flammable ecosystems and demonstrate the need to integrate historical and cultural legacies with ecological research (Foster et al. 2003).

**Figure 1:**
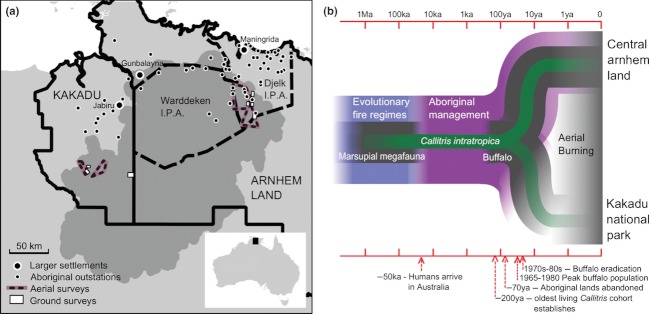
(a) Map of the Arnhem Plateau region illustrating the extent of sandstone outcrops comprising the Stone Country (shaded dark gray), settlements, and sampling areas. (b) Timeline showing the historical divergences in ecological conditions and management between Kakadu National Park and central Arnhem Land.

The Arnhem Plateau in northern Australia provides a compelling case study of the collision and integration of European and Aboriginal fire management (Fig. 1a). Colloquially referred to as the ‘Stone Country’, the 23,000-km^2^ sandstone massif is located in one of the world's most flammable landscapes (e.g., 1- to 3-year fire return intervals; Russell-Smith et al. 2003; Yates et al. 2008), straddling the border of World Heritage Kakadu National Park (KNP) and Arnhem Land. A millennial-scale history of fire management by Bininj-Kunwok Aboriginal clans was only recently disrupted by European expansion into the region at the turn of the 20th Century. The Stone Country is a conservation priority with high levels of species endemism and vast tracts of unmodified tropical savanna (Woinarski et al. 2006). However, the increasing prevalence of high-intensity wildfires, associated with the cessation of patchy landscape burning by Aborigines, has been linked to recent declines of fire-sensitive plant communities, mammals, and granivorous birds across the region (Bowman and Panton 1993; Franklin et al. 2005; Edwards and Russell-Smith 2009; Woinarski et al. 2010; Russell-Smith et al. 2012). Yet, despite attempts to ‘reimpose’ traditional burning by contemporary, institutionalized programs, as exemplified in KNP (Kakadu National Park Board of Management 2007), continued species declines contrast with high ecological integrity among areas still under Aboriginal management (Yibarbuk et al. 2001; Whitehead et al. 2003; Franklin et al. 2008), particularly in central Arnhem Land (CAL). We suggest that critical differences between KNP and CAL provide a unique natural experimental setting in which to understand complex social–ecological interactions involving fire, human history, megafauna, and cultural perceptions that would be impossible to manipulate, especially at large spatial scales (Diamond 1986). Our objective is, therefore, to provide a landscape scale contrast between KNP and CAL in order to understand how history and culture have shaped current management regimes and, consequently, savanna heterogeneity and diversity in a region globally renowned for its biology and culture, yet faced with daunting challenges in sustaining these values.

### Fire, megafauna, and humans on the Stone country

The Arnhem Plateau is over a billion years old, yet fire probably became prevalent in the region with the strengthening of the Asian Monsoon c. 20 Ma (Bowman et al. 2010). Evolutionary fire regimes likely consisted of less frequent, more intense fires than at present with higher forest cover (Kershaw et al. 2002). Climatic shifts throughout the Quaternary altered vegetation and fire, yet arguably the greatest ecological disruption in Australia occurred 40–60 ka with the largely coeval arrival of humans, extinction of megafauna, and a hypothesized increase in landscape burning (Jones 1969; Flannery 1990; Head 1996; Johnson 2009; Rule et al. 2012). The paleoecological evidence for changes in fire regime around this time remains equivocal (Kershaw et al. 2002; Mooney et al. 2011). However, the potential impacts of these changes on ecosystem composition is supported by contemporary research on the coupled effects of fire and megafaunal grazing (Knapp et al. 1999; Archibald et al. 2005; Waldram et al. 2008; Fuhlendorf et al. 2009) and the deliberate use of fire by indigenous people worldwide (Laris 2002; Bowman et al. 2011; Archibald et al. 2012).

The Bininj-Kunwok clans of the Stone Country maintain one of the world's oldest continuous cultures and few debate that Aboriginal burning is adaptive and ancient (Russell-Smith et al. 1997; Yibarbuk et al. 2001; Murphy and Bowman 2007). Sadly, the degradation of this tradition defines the region's next major ecological shift. European expansion into northern Australia in the late 19th Century devastated Aboriginal culture through disease and dispossession, leading to widespread abandonment of the Stone Country by the 1940s and 1950s (Cooke 2009). Consequently, fire regimes shifted from fine-scale burning by a widely dispersed population to high-intensity wildfires ignited largely in the late dry season by lightning (Ritchie 2009). Yet, the abandonment of the Stone Country was not ubiquitous. The plateau's eastern flank in CAL has remained under nearly continuous Aboriginal management (Yibarbuk et al. 2001). Scattered among more than a dozen outstations (small, family-based settlements) on ancestral lands, the Aboriginal population of the CAL Stone Country is smaller, more sedentary, and more dependent on imported goods than before European contact. However, the continuation of land management traditions is clear, with evidence of healthier lifestyles tied to subsistence resource extraction among outstation residents (Johnston et al. 2007; Garnett et al. 2009) and high ecological integrity of savannas on Aboriginal lands (Yibarbuk et al. 2001; Whitehead et al. 2003; Franklin et al. 2008).

The colonial introduction of a novel mega-herbivore, the Asian water buffalo (*Bubalus bubalis* L.), has further crystallized contrasts both in cultural perspectives and ecological conditions in KNP and CAL. Expanding from a few individuals in the mid-1800s to widespread populations within decades, buffalo became an icon of the Australian ‘frontier’, providing a common enterprise for white settlers and Aborigines via a hide industry that thrived until the 1950s (Bradshaw et al. 2007; Petty et al. 2007). As the industry declined, buffalo populations irrupted from 1960 to 1980, leading to severe impacts in wetlands and rainforests due to trampling and grazing (Werner et al. 2006; Petty et al. 2007). Yet, as their range expanded, Aboriginal Traditional Owners (TOs) integrated buffalo into their cosmology and subsistence resource base (Bowman and Robinson 2002; Robinson and Whitehead 2003). An eradication campaign targeting KNP, but not Arnhem Land, was eventually initiated, largely to protect the regional cattle industry from disease. Although buffalo impacts were and remain a concern to both European and Aboriginal land managers (Petty et al. 2007; Ens et al. 2010), the new perspective of buffalo as an environmental menace polarized many. Significantly, many TOs still viewed buffalo as a cultural component of the landscape and strongly disagreed with full-scale eradication (Haynes 2009). Buffalo densities in KNP were eventually driven to <0.01 km^−2^; however, populations in Arnhem Land were not targeted and continue to immigrate to the park (Robinson and Whitehead 2003; Petty et al. 2007).

### Divergent Stone Country management paradigms

The Stone Country's ecological history shows clear divergences in patterns of human occupancy and the institutionalization of land management (Fig. 1b). Established under a mandate of joint management in 1979, KNP's regulation of landscape burning and the highly contentious buffalo eradication program have led to conflict between TOs and park officials (Lewis 1989; Bradshaw et al. 2007; Trigger 2008; Haynes 2009). The current strategy for fire management in KNP's Stone Country (as well as western Arnhem Land) is, explicitly, an interpretation of Aboriginal burning imposed on a wilderness area (Kakadu National Park Board of Management 2007). Frequent fires are ignited in the early dry season, largely from helicopter, to disrupt fuel continuity, maintain habitat mosaics, and limit the extent of high-intensity fires in the late dry season. In practice, and largely out of necessity given limited resources and the landscape's size (e.g., one ranger, one helicopter for >5000 km^2^ in KNP), the program focuses on creating fire breaks along topographic features such as rivers and ridgelines, as opposed to patch mosaic burning *per se*. Park policy also imposes strict seasonal burning cut-offs to minimize the risk of late-season fires.

In contrast in Arnhem Land, the ‘outstation movement’ of the 1970s established small Aboriginal settlements, facilitating the management of ancestral lands by TOs. Although centralized Aboriginal Ranger groups have recently implemented aerial burning in CAL, the communities on the Stone Country's eastern flank remain a stronghold for Aboriginal culture (Yibarbuk et al. 2001). Thus, to the west is a highly institutionalized park system managing a wilderness area both for tourism and species conservation through regulated fire management and systematic buffalo eradication, while to the east is one of the most culturally intact social–ecological systems left in Australia, in which landscape burning and buffalo remain closely integrated with Aboriginal livelihoods. Aboriginal lands in CAL have been found to have high ecological integrity at the local scale (Yibarbuk et al. 2001; Whitehead et al. 2003) and, at the regional scale, more evenly distributed fire ignitions throughout the dry season compared with the early-season burning models under European tenure (Petty and Bowman 2007; Franklin et al. 2008). Thus, if the contrasting management paradigms in the KNP and CAL Stone Country affect the ecology of the region differently, then it should be possible to identify an ecological signal, despite the ‘noise’ associated with biophysical variation across the landscape. Here, we consider the biogeographical patterns of *Callitris intratropica* R.T. Baker & H.G. Smith and feral buffalo in order to examine ecological variation across the Stone Country and discuss the region's ecological and management trajectories.

## Methods

### *Callitris intratropica* as a Witness Tree

*Callitris intratropica*, one of the savanna's few non-eucalypt overstorey trees, is a fire-sensitive, Gondwanan cypress, which has provided insight into ecological change at regional and continental scales (Bowman and Panton 1993; Prior et al. 2011). Importantly, the life histories of extant *C. intratropica* individuals (>200 years) in northern Australia span the decline in Aboriginal burning and the introduction of buffalo. TOs have long recognized declines in *C. intratropica* as a signal of destructive fire regimes due to the species' vulnerability to intense fires, inability to resprout after burning, and longevity of dead stems (Haynes 1985). Widespread mortality in *C. intratropica* across northern Australia provides clear evidence of fire regime change (Bowman and Panton 1993; Edwards and Russell-Smith 2009; Russell-Smith et al. 2012). Closed-canopied groves of *C. intratropica* can also exclude low-intensity savanna fires via fuel suppression and maintain small (e.g., 0.02–0.5 ha), compositionally distinct forest patches within the savanna matrix. Yet, among open-canopied, degraded groves, flammability and plant community structure are identical to open savanna conditions, despite the persistence of *C. intratropica* adults (Trauernicht et al. 2012). The clear distinction between intact and degraded groves allows for rapid evaluation of the likelihood of groves to exclude fires. Thus, the condition of *C. intratropica* groves across the landscape can indicate both the prevalence of high-intensity fires as well as habitat heterogeneity not apparent from assessments of *C. intratropica* mortality alone.

### Plant community surveys

We surveyed the woody plant community associated with *C. intratropica* groves at 102 sites across the KNP and CAL Stone Country, restricting sampling to open, *Eucalyptus tetrodonta*/*E. miniata* savanna. Further logistic constraints included site access via seasonally flooded 4 × 4 tracks, multi-day walks, and permission from Aboriginal landowners. Survey sites consisted of discrete groves of *C. intratropica*, ranging in size from 100 to 3000 m^2^, located >500 m from one another. At each site, we randomly selected a reproductive *C. intratropica* adult within 5 m of the grove center and a reproductive *Eucalyptus miniata* or *E. tetrodonta* individual 30–100 m from the grove edge. Each tree marked the center of a 25-m^2^ circular plot in which we recorded canopy cover at the plot center using a spherical densiometer, the count and species of all woody plants >50-cm tall and the count of all *C. intratropica* seedlings <50-cm tall. At each plot, percent cover and mean height of graminoid fuels was measured in a randomly placed, 1 × 1 m^2^ quadrat. In addition, we scored the presence–absence of buffalo tracks as well as bowers of the great bowerbird (*Chlamydera nuchalis*) within or adjacent to each 25-m^2^ circular plot.

### Aerial surveys

Surveys of *C. intratropica* grove condition were conducted by helicopter across the KNP and CAL Stone Country (Fig 1a). As with ground surveys, we stratified sampling to open sandsheet savanna dominated by *E. tetrodonta* and *E. miniata*, avoiding rocky outcrops that afforded protection from fires. Each of the 33 groves in KNP and 54 in CAL were circled by the pilot while observers recorded (1) population structure by estimating the proportion of trees in each of three height classes relative to the established adult canopy (c. 15 m): saplings (1- to 3-m tall), juveniles (3- to 6-m tall), adults (>6-m tall), as well as dead adult trees; (2) shrub understory as absent, partial, or continuous; (3) lowest mean height of *C. intratropica* foliage (indicating prior canopy scorching); (4) buffalo track density as absent, few, or many; and (5) *Sorghum brachypodum* (a native species currently driving ecologically destructive grass-fire cycles in KNP) cover as low, moderate, or high.

### Analyses

In order to assess each *C. intratropica* grove sampled in the aerial surveys, the relevant attributes described above were weighted as 0, 0.5, or 1, with a higher weight indicating better ecological condition (Table 1). Attribute weights were added to derive a condition score for each grove, ranging from 0 (highly degraded) to 5 (highly intact). We examined the distributions of grove scores in KNP and CAL to compare the condition of *C. intratropica* between these management areas and statistically examined the effect of management on grove score using a simple least-squares linear model (‘lm’ function in R software; R Core Team) with a log-transformed response variable. We also separately modeled the effect of management area on aerial assessments of buffalo track density (absent/moderate/high) using ordinal logistic regression and *S. brachypodum* cover (low/moderate/high) using contingency analysis.

**Table 1:** The aerial survey observations, scored for each *Callitris intratropica* grove

Score	% Saplings	% Juveniles	% Dead trees	Foliage Ht	Shrub Cover
0	0	0	>20	>4 m	Absent
0.5	≤20	≤20	≤20	3–4 m	Partial
1	>20	>20	0	<3 m	Continuous

At each grove, we measured the percentage (relative to the total population) of (i) *C. intratropica* saplings (1–3 m), (ii) juveniles (3–6 m), and (iii) dead adult stems, as well as (iv) the mean lowest height of the canopy foliage (i.e., scorch damage) and (v) the cover of the understory shrub community. Scores were added to determine a total score for grove condition ranging from 0 (highly degraded) to 5 (highly intact).

For the ground survey data, we used the same criteria above to classify groves as either intact (grove score 3–5) or degraded (grove score 0–2). Thus, our primary explanatory variable, ‘plot type’, categorized plots as within intact *C. intratropica* groves, degraded *C. intratropica* groves, or *E. tetrodonta*/*miniata* savanna. Wherever possible, analyses employed multi-model inference using the Akaike Information Criterion based on the Information-Theoretic approach (Anderson and Burnham 2002). Unless stated otherwise, we used linear mixed models (LMM; ‘lme’ in R; Pinheiro et al. 2010) for variables with normally distributed errors and generalized linear mixed models (GLMM; ‘glmer’ in R; Bates et al. 2011) for variables with binomial- or Poisson-distributed errors, with site as a random factor.

To examine the woody plant community associated with *C. intratropica* groves, we calculated the frequency of occurrence for the top 25 most frequent species among intact groves, degraded, groves, and eucalypt savanna. We also examined the importance of *C. intratropica* groves to savanna gamma diversity by compiling species accumulation curves using random sampling from three different pools of plot types: (1) eucalypt plots only; (2) eucalypt and degraded *C. intratropica* plots only; and (3) eucalypt and intact *C. intratropica* plots only. To examine structural differences in the woody plant community, we modeled the count of all non-*C. intratropica* woody plant species as a function of plot type and the presence–absence of buffalo tracks using a log-normal Poisson GLMM for overdispersed data. *C. intratropica* seedling counts were modeled similarly. In order to examine the association of other fire-sensitive species with *C. intratropica* stands, we determined the proportion of obligate-seeder species and stems per plot (i.e., plants that do not resprout after burning; Northern Territory Ecological Response Database, J. Russell-Smith et al., unpubl. data). We then modeled each of these proportions as a function of plot type using binomial GLMMs. In order to corroborate buffalo observations between the aerial and ground surveys, we modeled the presence–absence of buffalo tracks in plots as a function of management area and plot type using a binomial GLM. The effects of plot type on percent graminoid cover and graminoid height were modeled using a binomial GLMM for proportional data and LMM, respectively. Finally, in order to further explore the effects of buffalo in this system, we revisited an analysis on the probability of savanna burning as a function of canopy cover – driven by *C. intratropica –* conducted in CAL (Trauernicht et al. 2012) and included the presence–absence of buffalo tracks in the global model.

## Results

**Figure 2:**
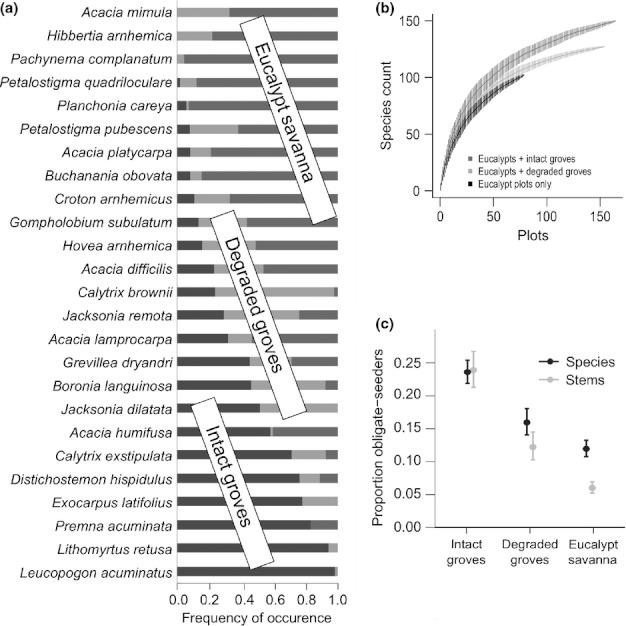
Woody plant species composition recorded in plots (N = 197) among intact and degraded *Callitris intratropica* groves as well as adjacent eucalypt savanna. (a) Frequencies among plot types for the 25 most frequent species of shrubs and small trees. (b) Species accumulation curves by random sampling from plots in eucalypt savanna and intact *C. intratropica* groves, eucalypt savanna and degraded *C. intratropica* groves, and eucalypt savanna only. (c) Proportions of species and stems of fire-sensitive, obligate-seeding plants (i.e., do not resprout after burning) sampled in each plot type (Akaike weight=0.99 relative to the null model for both analyses; error bars represent standard errors).

**Figure 3:**
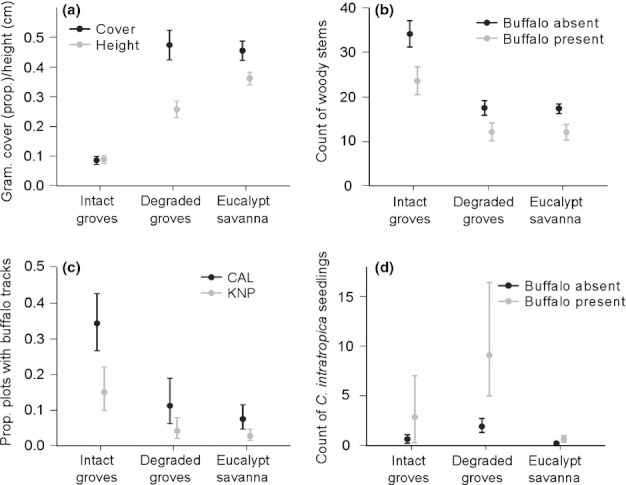
Comparisons among plots (*N* = 197) in intact and degraded *Callitris intratropica* groves and the surrounding eucalypt savanna matrix for (a) graminoid fuels height and cover (Akaike weight (w_i_)=0.99, both analyses), (b) the count of woody plants (excluding *C. intratropica*) including the effect of the Asian water buffalo (w_i_=0.92), (c) the proportion of plots containing buffalo tracks in central Arnhem Land (CAL) and Kakadu National Park (KNP; w_i_ = 0.79), and (d) the count of *C. intratropica* seedlings including the effect of buffalo (w_i_ = 0.97). Akaike weights are presented relative to the null model and error bars represent standard errors.

**Figure 4:**
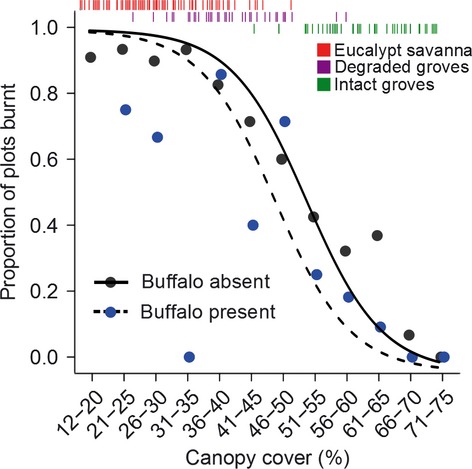
The probability of savanna burning as a function of canopy cover and the presence–absence of buffalo tracks (*N* = 421, Akaike weight=0.76, *R*^*2*^ = 0.30). Rug plots at the top of the figure indicate canopy cover distributions among intact and degraded *Callitris intratropica* groves and the adjacent eucalypt savanna.

The frequency of occurrence of plant species illustrated a distinct community association with intact *C. intratropica* groves (Fig. 2a). Plotting species accumulation curves using plots in eucalypt savanna and intact *C. intratropica* groves increased woody plant richness beyond that sampled in eucalypt plots alone, while including degraded stands had a much smaller effect on richness (Fig. 2b). The plant community within intact *C. intratropica* groves also had significantly higher proportions of species and stems of obligate-seeding plants (i.e., species do not resprout after fire and decline under high fire frequencies; Edwards and Russell-Smith 2009; Russell-Smith et al. 2012) than the eucalypt savanna (Fig. 2c). Interestingly, 30% of the intact *C. intratropica* groves surveyed in KNP contained greater bowerbird (*Chlamydera nuchalis*) bowers, while none were encountered in other plot types. In terms of plant community structure, our statistical models suggested that *C. intratropica* patch dynamics (Trauernicht et al. 2012) extend across the landscape, with lower graminoid (i.e., grassy fuel) cover and height (Fig. 3a) and higher woody plant density among intact groves, but that buffalo reduced woody stem counts (Fig. 3b). Our results also demonstrated a clear association between buffalo and intact *C. intratropica* groves (Fig. 3c) and the presence of buffalo tracks was associated both with higher counts of *C. intratropica* seedlings and a lower probability fire occurrence (Figs 3d, 4).

**Figure 5:**
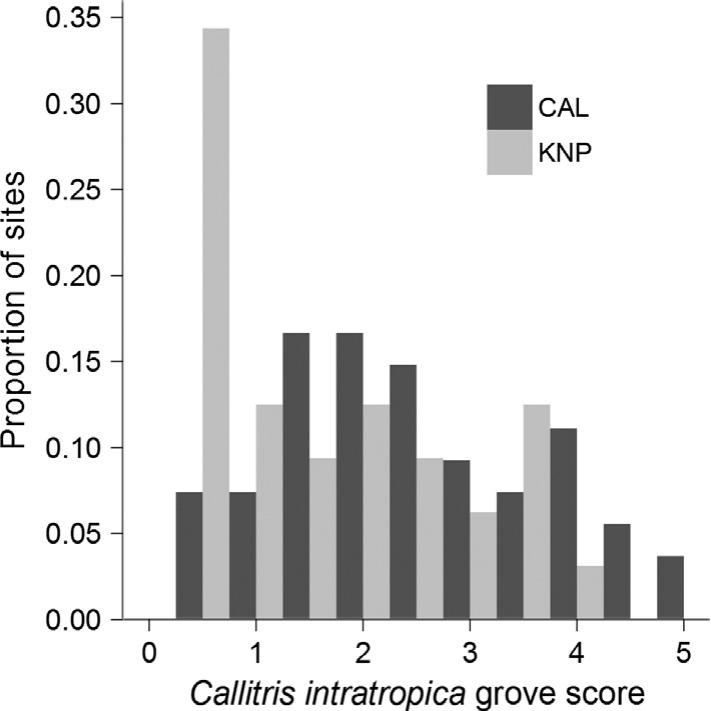
Distributions of *Callitris intratropica* grove condition scores in Kakadu National Park (KNP; *N* = 33) and central Arnhem Land (CAL; *N* = 54). Scores were derived from aerial surveys of population structure (including dead stems), the height of canopy scorching, and the cover of the understory shrub community. A least-squares linear model of log-transformed scores supported the importance of region (KNP vs. CAL) in explaining this difference (Akaike weight=0.99 relative to null model).

Scaling patch-level (i.e., individual grove) dynamics to the landscape scale (i.e., multi-kilometer transects; Fig. 1a) via aerial surveys, the distribution of grove condition scores clearly showed a higher proportion of intact *C. intratropica* groves in CAL (Fig. 5). Aerial surveys also confirmed higher buffalo densities in Arnhem Land noted in other studies (Koenig et al. 2003; Franklin et al. 2008) with 26% of sites in CAL containing moderate to high buffalo track density vs. <1% in KNP (χ^2^ = 6.09, d.f. = 1, P = 0.014;). Aerial surveys indicated low *S. brachypodum* cover at all our CAL sites versus 22% and 53% of sites in KNP with partial and high cover, respectively (χ ^2^=56.2, d.f. = 2, P < 0.0001).

## Discussion

The results of our ground surveys demonstrate that the presence of intact *C. intratropica* groves within open eucalypt savanna increases plant gamma diversity and structural complexity across the landscape. Reaching densities of more than one per hectare (C. Trauernicht, unpubl. data), *C. intratropica* groves clearly provide refugia for fire-sensitive, obligate-seeding plants and at least one bird species within the matrix of more open, fire-adapted vegetation. Thus, the higher proportion of intact *C. intratropica* groves in the CAL Stone Country recorded from aerial surveys (Fig. 2) not only suggests a lower prevalence of high-intensity fires but also that the savanna in CAL is more structurally and biologically diverse than KNP. Given the differences in ecological history between these regions (Fig. 1b), our results implicate the role of Aboriginal management in shaping patterns of vegetation, and suggest that there is a relationship between cultural and ecological integrity in these savannas. Previous research has demonstrated the ecological benefits of Aboriginal management at local scales (Yibarbuk et al. 2001; Whitehead et al. 2003; McGregor et al. 2010). Our findings suggest that these benefits may extend to the landscape scale given the spatial extent of our surveys across a large area of CAL Stone Country accessed and utilized regularly by TOs via multiple outstations (Fig. 1a). Furthermore, it is important to note that the presence of degraded *C. intratropica* groves in KNP implies that conditions there were once very similar to CAL. Evidence suggests the process of grove degradation and, more generally, the homogenization of savanna vegetation are unidirectional processes (Russell-Smith et al. 2012; Trauernicht et al. 2012). Thus, based on intact KNP grove composition, we can infer that degraded groves in KNP once also contributed to the heterogeneity and diversity of savanna vegetation. The patch dynamics of *C. intratropica* therefore provide a powerful tool for understanding the patterns and processes of landscape change in the Stone Country.

### People and buffalo as ecosystem engineers?

We suggest that in addition to differences in Aboriginal occupation, the control of feral buffalo has had major effects on patterns of landscape burning across the Stone Country. Megafaunal grazers have been described as ecosystem engineers based on their strong effects on herbaceous vegetation and the coupling of these impacts with fire disturbance (Knapp et al. 1999; Waldram et al. 2008; Fuhlendorf et al. 2009). Our results for fire occurrence and *C. intratropica* recruitment (Figs 3d and 4) suggest that buffalo are interacting similarly in the Stone Country. This indicates that the compartmentalization of buffalo and fire as separate phenomena by contemporary management programs may be misguided, certainly according to the cultural perspectives of TOs (Bowman and Robinson 2002; Robinson and Whitehead 2003; Trigger 2008), and also ecologically.

Although buffalo–fire interactions may benefit *C. intratropica*, the hypothesis that buffalo may be beneficial to the Stone Country savannas is likely to produce contentious debate. Outside wetlands, however, little vegetation change was observed in the KNP Stone Country over the course of buffalo expansion and decline (Petty et al. 2007). Further landscape-scale assessments in KNP have found little to no effect of buffalo density on tree dynamics in upland savannas (Bowman et al. 2008; Lehmann et al. 2008), whereas at the site level, buffalo grazing has been shown to decrease fire damage and mortality among woody savanna species (Werner 2005; Werner et al. 2006). Thus, although critical wetland habitats certainly require protection (Petty et al. 2007; Ens et al. 2010), we are far from a complete understanding of buffalo interactions in the system. Ultimately, given the high costs and logistic difficulties of eradication, the Aboriginal view of buffalo as an active ecological component of these savannas likely provides a more realistic perspective for management.

### Hybrid land management – the way forward?

Contemporary Stone Country burning programs were only established after extensive fires in 2004 and 2006 and the region presents myriad challenges. For instance, with one ranger handling KNP's program, covering the entire landscape during the early dry season is difficult, even by helicopter, and limits the opportunities for TOs to burn on the ground (although several ‘footwalks’ are organized each year; A. Pickworth, pers. comm.). KNP is simultaneously saddled with huge tourism responsibilities and managing both objectives stretches the limits of funding and human resources. East of KNP, the Western Arnhem Land Fire Abatement project (WALFA) provides yet another model of joint management for the Stone Country (Whitehead et al. 2009). WALFA is funded through carbon offsets for corporations, employing aerial and ground burns in the early dry season to reduce biomass consumed by late dry season fires (Russell-Smith et al. 2009). Among WALFA's major achievements has been the establishment of the Warddeken Ranger group, providing access and culturally appropriate employment to TOs in one of the most remote regions of the Stone Country. Similarly, the Djelk Ranger group has augmented Aboriginal participation in land management in CAL in addition to the activities of outstation residents. Although these programs stand at the forefront of progressive fire management, the reciprocity among differing objectives – carbon offsets, biodiversity conservation, cultural identity, etc. – is still unresolved and leaves potential both for collaboration and conflict (Andersen 1999; Yibarbuk et al. 2001; Russell-Smith et al. 2003).

Feral buffalo may present even greater controversy. Although we cannot extract the ways in which buffalo effects confound the influence of people on savanna integrity from our data, we argue that the interactions between buffalo and fire is an implicit component of contemporary Aboriginal land use, in which the species is valued and managed for hunting (Bowman and Robinson 2002). Given the fact that eradication is, in the end, financially unsustainable, it seems far more productive to examine the role of grazing as a management tool. In KNP, the grass–fire cycle involving the native annual *Sorghum brachypodum* provides a prime example where continuous, monotypic fuel beds of the species in many areas drive more intense, large-scale fires (Elliott et al. 2009). Although the same species is present in Arnhem Land, aerial surveys indicated low *S. brachypodum* cover at all CAL sites versus high cover at over half the sites in KNP. High numbers of buffalo are clearly ecologically destructive (Petty et al. 2007; Ens et al. 2010) – even among *C. intratropica* groves (Fig. 4b) – however, targeted stocking rates may provide both a tool with which to combat current and impending grass–fire cycles as well as the opportunity to engage TOs.

A legacy of research in Australia has pioneered the ecological significance of humans in flammable systems (Jones 1969; Bowman and Panton 1993; Head 1996; Russell-Smith et al. 1997; Yibarbuk et al. 2001; Whitehead et al. 2003; Murphy and Bowman 2007; Bird et al. 2008; Fletcher and Thomas 2010), a dynamic that is increasingly apparent elsewhere (Laris 2002; Rodriguez 2007; Archibald et al. 2012). Although it is difficult to ascertain the degree of replicability or consistency in land use among outstations, the greater heterogeneity and diversity in the CAL Stone Country indicated by *C. intratropica* (Fig. 5) suggests an association between greater ecological integrity and a more holistic, culturally based approach to land management. Our findings also contribute to the current debate over the ecological benefits of landscape heterogeneity wrought by fire, or ‘pyrodiversity’ (Parr and Andersen 2006; Parr and Brockett 2008). The persistence of *C. intratropica* groves in these savannas is clearly tied to the maintenance of lower intensity, patchier fires (Bowman and Panton 1993; Edwards and Russell-Smith 2009; Russell-Smith et al. 2012; Trauernicht et al. 2012). Thus, the contribution of intact *C. intratropica* groves to savanna plant species richness – particularly the occurrence of fire-sensitive species (Fig. 2) – provides a clear link between pyrodiversity and biodiversity.

Equally compelling as the potential ecological benefits, TOs living ‘on country’ enjoy greater mental and physical well-being than those in larger settlements, as unequivocally demonstrated by Aboriginal testimony and socio-medical research (Johnston et al. 2007; Garnett et al. 2009). The scenarios in CAL and WALFA suggest that increasing and/or reintegrating the direct involvement of TOs in land management is a tenable goal. Although we and others contend that the contemporary institutions like KNP must prioritize the rehabilitation of Aboriginal lands as social–ecological systems (Altman and Whitehead 2003; Whitehead et al. 2003; Johnston et al. 2007), many barriers persist: sociopolitical opposition to outstations, loss of traditional knowledge, and limited access to ancestral lands are but a few. Buffalo will also remain a flashpoint for conservationists, yet similarly, burning restrictions and tourism may not align with the wishes of TOs. The story of people, fire, and buffalo on the Stone Country affirms the resilience and adaptability of a culture that has persisted for more than 50 millennia. Clearly, the sustainability of the Stone Country, as well as other cultural landscapes, depends upon sustainable futures for indigenous people. This challenges all actors to confront conflicting needs and perspectives, yet also finds common ground, in order to develop and improve management paradigms.

## Biosketches

Clay Trauernicht is a PhD candidate in the School of Plant Science at University of Tasmania. His research interests include coupled social–ecological systems and the relationships between ecosystem dynamics, management, and human welfare at community and landscape scales.
